# Dually Active Polycation/miRNA Nanoparticles for the Treatment of Fibrosis in Alcohol-Associated Liver Disease

**DOI:** 10.3390/pharmaceutics14030669

**Published:** 2022-03-18

**Authors:** Chuhan Zhang, Yu Hang, Weimin Tang, Diptesh Sil, Heather C. Jensen-Smith, Robert G. Bennett, Benita L. McVicker, David Oupický

**Affiliations:** 1 Center for Drug Delivery and Nanomedicine, Department of Pharmaceutical Sciences, College of Pharmacy, University of Nebraska Medical Center, Omaha, NE 68198, USA; chuhan.zhang@unmc.edu (C.Z.); yuhang_93@163.com (Y.H.); weimin.tang@unmc.edu (W.T.); diptesh.sil@unmc.edu (D.S.); 2 Eppley Institute for Cancer Research & Fred and Pamela Buffer Cancer Center, University of Nebraska Medical Center, Omaha, NE 68198, USA; heather.jensensmith@unmc.edu; 3 Department of Internal Medicine, University of Nebraska Medical Center, Omaha, NE 68198, USA; rgbennet@unmc.edu (R.G.B.); bmcvicker@unmc.edu (B.L.M.); 4 Department of Biochemistry and Molecular Biology, University of Nebraska Medical Center, Omaha, NE 68198, USA; 5 VA Nebraska-Western Iowa Health Care System, Omaha, NE 68105, USA

**Keywords:** nanoparticles, miR-155, CXCR4, alcohol-associated liver disease (AALD)

## Abstract

Alcohol-associated liver disease (AALD) is a major cause of liver disorders worldwide. Current treatment options are limited, especially for AALD-associated fibrosis. Promising approaches include RNA interference for miR-155 overexpression in Kupffer cells (KCs), as well as the use of CXCR4 antagonists that inhibit the activation of hepatic stellate cells (HSCs) through the CXCL12/CXCR4 axis. The development of dual-functioning nanoparticles for the effective delivery of antifibrotic RNA together with a CXCR4 inhibitor thus promises to improve the treatment of AALD fibrosis. In this study, cholesterol-modified polymeric CXCR4 inhibitor (Chol-PCX) was synthesized and used to encapsulate anti-miR-155 or non-coding (NC) miRNA in the form of Chol-PCX/miRNA nanoparticles. The results indicate that the nanoparticles induce a significant miR-155 silencing effect both in vitro and in vivo. Treatment with the Chol-PCX/anti-miR-155 particles in a model of moderate alcohol consumption with secondary liver insult resulted in a significant reduction in aminotransferase enzymes as well as collagen content in the liver parenchyma. Overall, our data support the use of Chol-PCX as a carrier for anti-miR-155 for the combined therapeutic inhibition of CXCR4 and miR-155 expression as a way to improve fibrotic damage in the liver.

## 1. Introduction

Alcohol overuse causes damage to the liver and multiple other organs. Up to a third of people who misuse alcohol develop liver damage [1,2]. Long-term alcohol consumption causes chronic liver disease, including liver fibrosis, cirrhosis, and hepatocellular carcinoma [3]. There are no approved treatments for liver fibrosis, resulting in liver transplantation as the only curative option. The mechanisms underlying alcohol-associated liver disease (AALD) are complicated. Heavy alcohol use can affect fatty acid synthesis and oxidation [4,5]. Furthermore, alcohol-related changes in gut permeability increase portal vein endotoxins, leading to innate immune responses and liver inflammation through the activation of several cytokine cascades. Multiple hepatic cells are involved in modulating the complex liver microenvironment, including resident macrophages, Kupffer cells (KCs), hepatic stellate cells (HSCs), liver sinusoidal endothelial cells (LSECs), and hepatocytes [6]. A central event during AALD is the sensitization of KCs, resulting in the promotion of inflammatory factors and fibrogenesis rather than wound healing. The KC inflammatory response leads to the production of cytokines/chemokines and reactive oxygen species that promote the transformation of normally quiescent HSCs into an activated myofibroblast phenotype that facilitates the deposition of extracellular matrix (ECM) in addition to scar formation [7]. The events surrounding KC and HSC activities during AALD progression provide opportunities for targeted drug delivery to improve treatment.

Nanoparticle delivery systems have been developed for a range of liver diseases due to their tendency to accumulate in the liver [8,9]. However, very few nanoparticle delivery systems have been tested in alcohol-associated liver fibrosis models. Here, we are therefore mainly focusing on delivery carriers to AALD fibrosis. The use of nanoparticles for the treatment of AALD fibrosis is appropriate as nanoparticles are efficiently taken up by KCs followed by other hepatic cells, including LSECs and HSCs [10,11,12,13,14]. The predominant uptake in KCs is due to their phagocytic nature and portal location. To further enhance the specificity of nanoparticle delivery, specific ligands can be used to partially redirect uptake from KCs to other types of hepatic cells.

RNA interference (RNAi) is a promising method for treating various diseases, including cardiovascular disorders, cancers, and liver problems [15]. The biggest challenge to the use of RNAi is successful systemic delivery, because small RNAs, such as siRNA and miRNA, are unstable in serum and their highly negative charge makes crossing cell membranes difficult. However, nanoparticles can be used to encapsulate small RNAs to achieve targeted delivery to disease sites, thereby overcoming the obstacles of unencapsulated RNA [16]. MiRNAs are endogenous, small, and non-coding 21–23-nucleotide RNAs that participate in various regulatory mechanisms in liver diseases. They regulate gene expression by binding to the 3′ untranslated region of target mRNA to suppress translation or induce mRNA cleavage [17]. Among potential targets in AALD, miR-155 is involved in the pathogenesis of the disease, with reported upregulation in KCs and hepatocytes [18]. The therapeutic potential of the miR-155 pathway was demonstrated by reduced fat accumulation in miR-155 knockout (KO) mice through an increase in peroxisome proliferator hormone response elements binding with peroxisome proliferator-activated receptors in KCs [19]. Lipopolysaccharide (LPS) is recognized by the Toll-like receptor (TLR) 4 complex and is considered an important player in regulating inflammatory cytokine activation in the liver. Through continuous alcohol exposure, KCs are sensitized by LPS, which induces an inflammatory response. Thus, miR-155 knockdown can reduce alcohol-induced liver injury, steatosis, and inflammation by the inhibition of TLR4 signaling [20].

The activation of HSCs is an important event in the development and progression of fibrosis during AALD [21]. Among various chemokine receptors, CXC receptor 4 (CXCR4) and its ligand, CXCL12, are overexpressed in activated HSCs known to regulate pathogenic mechanisms. CXCR4 promotes the activation and differentiation of HSCs in liver fibrosis through MAPK activation [22]. Though there is no consensus on the role of CXCR4 in AALD fibrosis, there are studies showing that the blockading of CXCR4 signaling suppresses HSC activation and proliferation, leading to a downregulation of collagen I and α-SMA expression in liver fibrosis [22,23].

**Figure 1:**
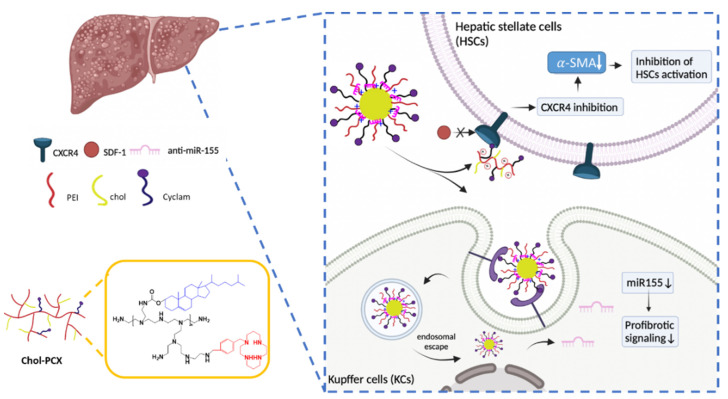
Schematic illustration of the mechanisms of the antifibrotic effect of Chol-PCX/anti-miR-155.

Our previous studies reported on the development of a series of polycationic CXCR4 antagonists (PCX) that deliver siRNA and miRNA in various disease models, including metastatic cancers, leukemia, and lung fibrosis [24,25,26]. Small-molecule CXCR4 antagonists, such as AMD3100, show therapeutic effects stemming from the inhibition of the CXCL12/CXCR4 axis [27]. Here, we hypothesized that nanoparticles consisting of the CXCR4-inhibiting PCX and encapsulated miRNA will be capable of therapeutically targeting both the KCs and activated HSCs in AALD. We describe the synthesis of cholesterol-modified PCX (PCX-Chol) based on polyethyleneimine (PEI) as a system to simultaneously target activated HSCs and KCs in a model of AALD fibrosis. Figure 1 illustrates the proposed mechanism of action of the PCX system for the delivery of anti-miR-155. This study characterized the effect of nanoparticles on miR-155 and CXCR4 expression as well as therapeutic outcomes in a model of hepatic fibrosis during AALD.

## 2. Materials and Methods

### 2.1. Materials

Cyclam (1,4,8,11-tetraazacyclotetradecane) was purchased from Sigma-Aldrich (St. Louis, MO, USA). Branched PEI (10 kDa) was purchased from Polysciences (Warrington, PA, USA). Solvents (certified ACS grade) were purchased from Fisher and used without further purification. miRIAN microRNA hairpin inhibitor (anti-miR-155) and miRIDIAN microRNA hairpin inhibitor negative control (anti-miR-NC) were bought from Horizon (Perkin Elmer, UK). Dulbecco’s phosphate-buffered saline (PBS), trypsin, penicillin/streptomycin (Pen-Strep), Dulbecco’s modified Eagle medium (DMEM), and fetal bovine serum (FBS) were from Hyclone (Waltham, MA, USA). Human CXCL12 was bought from Shenandoah Biotechnology Inc. FAM^TM^ Dye-Labeled anti-miR^TM^ Negative Control was purchased from Fisher. Cy5.5-siRNA was purchased from Dharmacon^TM^. Lipopolysaccharides (LPS) from *Escherichia coli* O127:B8 were bought from Sigma. CXCR4, type-I collagen (Col1a1), matrix metalloproteinases (MMPs), and tissue inhibitors of metalloproteinases (TIMPs) primers were bought from Sigma.

### 2.2. Synthesis and Characterization of Chol-PCX

PEI (64.65 mg), tri-tert-butyl-11-(4-(chloromethyl)benzyl)-1,4,8,11-tetraazacyclotetradecane-1,4,8-tricarboxylate (772 mg, 1.2 mmol) (synthesized as previously described [28]), and potassium carbonate (415 mg, 3 mM) were suspended in acetonitrile (20 mL) and refluxed for 16 h, followed by filtration and solvent evaporation to obtain PCX. The PCX (100 mg) was then dissolved in a mixture of anhydrous methylene chloride and *N,N*-diisopropylethylamine. Cholesteryl chloroformate (15 mg, 3.3 mM) in anhydrous methylene chloride was added dropwise over 1 h. The reaction was continued under stirring for another 24 h. The product was obtained by evaporating the solvent and washing with diethyl ether three times to remove the unreacted cholesteryl chloroformate. Trifluoroacetic acid (20 mL) was added and the mixture was stirred overnight to remove the t-butyloxycarbonyl (Boc) protecting groups prior to dialysis against distilled water and lyophilization to obtain Chol-PCX. The ^1^H NMR spectrum of Chol-PCX in DMSO-d_6_ was recorded on a Bruker 500 MHz NMR spectrometer at room temperature.

### 2.3. Preparation and Characterization of PCX/miRNA Nanoparticles

The nanoparticles were prepared by mixing equal volumes of miRNA and Chol-PCX solutions (10 mM HEPES, pH 7.4) to achieve the desired polymer/RNA *w*/*w* ratios. For *w*/*w* 4, 0.5 mL of a 40 μg/mL miRNA solution was mixed with a 160 μg/mL Chol-PCX solution to a final 20 μg/mL miRNA concentration. After being vigorously vortexed for 10 s and incubated at room temperature for 30 min, the particles were characterized. The hydrodynamic particle size and zeta potential were measured by dynamic light scattering (DLS) at 25 °C using a Malvern NANO ZS (Cambridge, UK).

### 2.4. Cell Viability and Intracellular Trafficking

Immortalized human HSC cells (LX-2 cells, received as a kind gift from Dr. Scott L. Friedman, Icahn School of Medicine at Mount Sinai [29]) were cultured under 5% CO_2_ in DMEM supplemented with 2% FBS and 1% Pen-Strep at 37 °C. Murine RAW 264.7 macrophages (ATCC, Manassas, VA, USA) were cultured under 5% CO_2_ in DMEM supplemented with 10% FBS and 1% Pen-Strep at 37 °C. When confluency reached 80–90%, the cells were trypsinized and subcultured.

Cell viability was evaluated by a CellTiterBlue assay following the manufacturer’s protocol (Promega Corp. Madison, WI, USA). In brief, 8000 cells/well were seeded in a 96-well plate and cultured for 20–24 h. A series of increasing concentrations of the polymers and nanoparticles were added, and the plates were incubated at 37 °C for 24 h. Cell viability was normalized to cells incubated with PBS by measuring the absorbance at 560_Ex_/590_Em_ nm using a SpectraMax iD3 Multi-Mode Microplate Reader.

For uptake evaluation, the cells (15,000 cells/well) were seeded in 12-well plates and cultured to 60% confluency. The cells were incubated with the nanoparticles containing fluorescently labeled FAM-miRNA (100 nM) for 4 h. Cellular uptake was measured in trypsinized cells using a BD FACS LSR II Green flow cytometer.

### 2.5. In Vitro miRNA Transfection

RAW 264.7 cells (2 × 10^5^ cells/well) were seeded in 6-well plates and cultured to 60% confluency. The cells were incubated with the nanoparticles (100 nM of anti-miR-155) or 100 nM of anti-miR-155/Lipofectamine 2000 as the positive control for 6 h in a serum-free medium, then removing the nanoparticle solutions followed by stimulation with LPS (100 ng/mL) for 24 h. RT-PCR analysis was performed using a Rotor-Gene Q (QIAGEN) thermocycler with a miRCURY LNA miRNA PCR Starter Kit (QIAGEN). The universal reverse transcription was followed by real-time PCR amplification with the LNA-enhanced primers. MiR-155 expression was expressed relative to the internal control UniSp6.

### 2.6. CXCR4 Antagonism Assay

The CXCR4 antagonism efficacy of Chol-PCX was determined by a CXCR4 redistribution assay. Human epithelial osteosarcoma U2OS cells with EGFP-CXCR4 fusion protein (Fisher Scientific Waltham, MA, USA) were cultured in DMEM supplemented with 2 × 10^−3^ M L-glutamine, penicillin (100 U/mL), streptomycin (100 μg/mL), G418 (0.5 mg/mL), and 10% FBS. The cells (8000 cells/well) were seeded in black 96-well microplates. After 24 h, the cells were washed with an assay buffer and treated with Chol-PCX at different concentrations for 30 min. AMD3100 (300 nM) was used as a positive control. The CXCL12 (10 nM) was added and incubated for 1 h. Cells were fixed and visualized using EVOS xl microscopy under the GFP channel.

### 2.7. In Vivo Anti-miR-155 Therapy in AALD Fibrosis

All animal experiments were performed in C57BL/6 female mice purchased from Jackson Laboratories and following the protocol approved by the University of Nebraska Medical Center Institutional Animal Care and Use Committee. An AALD fibrosis model was established by feeding the mice an EtOH-containing Lieber–DeCarli (LD) daily liquid diet (Dyets Inc., Bethlehem, PA, USA) combined with repeated intraperitoneal injection of CCl_4_ for 3 weeks, modified from a previous report [30]. In brief, on day 0 mice were started on 1% (*v*/*v*) EtOH LD liquid diet or control isocaloric liquid diet without EtOH for two days. Starting on day 3, the mice were injected twice per week with CCl_4_ (1 mL/kg of 10% CCl_4_ in olive oil) and fed with 2% EtOH LD liquid diet or the control isocaloric diet. The daily intake of the EtOH mice was monitored, and the following day the equivalent calories were administered to control mice. Starting the second week, mice were injected twice with the treatment or control nanoparticles. All nanoparticle treatments were given 24 h after a CCl_4_ injection. There were four treatment groups, with 6 mice per group: (i) pair-fed control, (ii) EtOH LD diet + CCl_4_ + PBS, (iii) EtOH LD diet + CCl_4_ + Chol-PCX/anti-miR-NC, and (iv) EtOH LD diet + CCl_4_ + Chol-PCX/anti-miR-155. Nanoparticles (10 μL/kg) were injected via tail vein at a dose of 1 mg/kg of miRNA and a polymer/miRNA ratio of 4. Mice were sacrificed 24 h after the last treatment and the liver tissues as well as the blood samples were collected. The livers were stored in RNAlater (Qiagen, Valencia, CA, USA) or 10% formalin. The mRNA expression of fibrotic markers was analyzed by SYBR Green RT-PCR. Extracted RNA (2 µg) was converted into cDNA using a High-Capacity cDNA Transcription Kit (Applied Biosystems, Waltham, MA, USA). The PCR reactions were run on Rotor-Gene Q (QIAGEN) equipment with iTaq Universal SYBR Green Supermix (Bio-Rad, Hercules, CA, USA) and GAPDH as a housekeeping gene. Relative mRNA levels were calculated based on the comparative threshold value (Ct) method. The serum concentration of alanine transaminase (ALT) and aspartate amino transaminase (AST) was determined by a VITROS 5.1 FS Chemistry System (Ortho Clinical diagnostics) at the Omaha VA Medical Center Clinical Chemistry Laboratory.

### 2.8. Biodistribution

AALD fibrosis in C57BL/6J mice was induced as stated in Section 2.7. Then, both the EtOH mice and pair-fed mice were injected intravenously with 200 μL of fluorescently labeled Cy3−Chol−PCX/Cy5.5−siRNA or Cy3−PEI/cy5.5−siRNA (1 mg/kg Cy5.5−siRNA, 4 mg/kg Cy3−Chol−PCX or Cy3−PEI). Mice were sacrificed 24 h post-injection, and major organs were harvested for imaging by Xenogen IVIS 200.

### 2.9. Histology and Immunohistochemistry

The liver specimens were fixed in 10% formalin overnight and then embedded in paraffin. Sections (5 µm) were stained with Sirius Red, hematoxylin and eosin (H&E), and Masson’s trichrome by the UNMC Tissue Sciences Facility. Brightfield images at low power (10× objective) were captured for at least 5 random fields per sample using a Nikon Eclipse 80i microscope and DS-Fi2 camera. Automated image analysis was performed in a blinded manner by using ImageJ software with the color deconvolution, background subtraction, and threshold (max entropy) functions [31,32,33].

For immunohistochemistry, formalin-fixed paraffin-embedded sections were dewaxed and subject to epitope retrieval by heating in a pressure cooker in 10 mM tris(hydroxymethyl)aminomethane buffer containing 1 mM ethylenediamine tetraacetic acid (EDTA) with pH 9.0 for 30 min, followed by cooling at room temperature for 30 min. Endogenous peroxidases were quenched for 10 min with Bloxall (Vectorlabs), followed by blocking in 5% normal goat serum for 30 min. Slides were incubated overnight at 4 °C with primary rabbit monoclonal antibodies specific for F4/80 (#70076, Cell Signaling Technology, 1:500), CD44 (#37259, Cell Signaling Technology, 1:400), smooth muscle actin (SMA, #19245, Cell Signaling Technology, 1:1000), or rabbit recombinant antibody specific for CD163 (#ab182422, Abcam, 1:2000), diluted in SignalStain Antibody Dilution Buffer (Cell Signaling Technology). After washing, slides were incubated for 30 min at room temperature with horseradish peroxidase polymer-conjugated anti-rabbit antibody (Cell Signaling Technology). The detection was carried out by tyramide signal amplification as described in [34], using AlexaFluor-647 or -488 tyramide (ThermoFisher), and slides were mounted in Prolong Gold with DAPI (ThermoFisher). Fluorescent micrographs were captured from low-power fields using a Nikon Eclipse 80i microscope and Cool SnapEZ camera (Photometrics). Automated image analysis was performed in a blinded manner with ImageJ software using the Threshold (Ohtsu) function.

### 2.10. Second Harmonic Generation (SHG) Imaging

The imaging of endogenous collagen by second-harmonic generation (SHG) was conducted in the Mutliphoton Intravital and Tissue Imaging (MITI) core at the UNMC using an upright Olympus FVMPE-RS microscope equipped with a Spectra-Physics InSight X3 laser and 25× (1.05 NA) objective. Images were collected from regions containing central venules using 860 nm excitation. SHG-specific emissions were collected in individual images taken at 1 µm intervals throughout each 5 µm section using a 432 nm (45 nm bandpass) emission filter and zoom of 1.2. To optimize the visualization of collagen fiber lengths, 5 µm image stacks were compressed using NIH ImageJ (max projection) [35]. Collagen fiber counts, organization (fiber length, width, and curvature), and alignment were subsequently quantified in individual SHG images (424 mm × 424 mm × 5 mm, 0.414 mm/pixel) using CT-FIRE and CurveAlign for fibrillar collagen quantification [36,37,38]. All images and subsequent analyses were conducted by a researcher blinded to the sample conditions.

### 2.11. Statistical Analysis

The results are presented as mean ± SD or SEM. One-way ANOVA was used and followed by Tukey’s multiple comparison test to analyze statistical differences among multiple groups. Differences were assessed to be significant: * *p* < 0.05 was considered as a minimal level of significance, and ** *p* < 0.01 and *** *p* < 0.001 were considered as very significantly different. All the statistical analyses were performed with GraphPad Prism 8.

## 3. Results

### 3.1. Preparation and Characterization of miRNA Nanoparticles

Chol-PCX was synthesized as previously described [39]. We used a synthetic approach that was designed to minimize the modification of the CXCR4-binding cyclam moieties with cholesterol by using the Boc protection of the cyclams. Thus, the cholesterol modification was primarily directed at the secondary amines of PEI (Figure 1).

**Figure 2:**
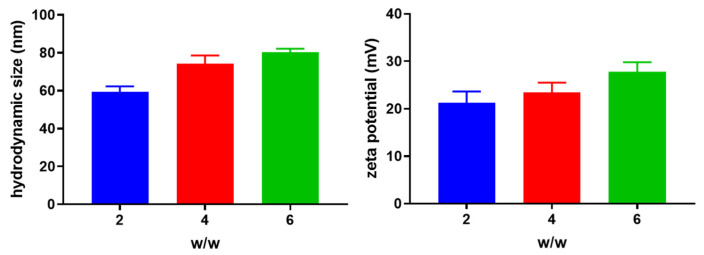
Characterization of Chol-PCX/miRNA nanoparticles. Hydrodynamic size and zeta potential of nanoparticles prepared at different Chol-PCX/miRNA *w*/*w* ratios.

The hydrodynamic size and zeta potential of Chol-PCX/miRNA nanoparticles prepared at different *w*/*w* ratios were measured using DLS. The particle size increased slightly from 60 to 80 nm with an increase in the polymer/miRNA *w*/*w* ratio (Figure 2). The zeta potential of the nanoparticles also increased slightly along with the enhanced *w*/*w* ratio.

### 3.2. Cytotoxicity and Intracellular Trafficking

**Figure 3:**
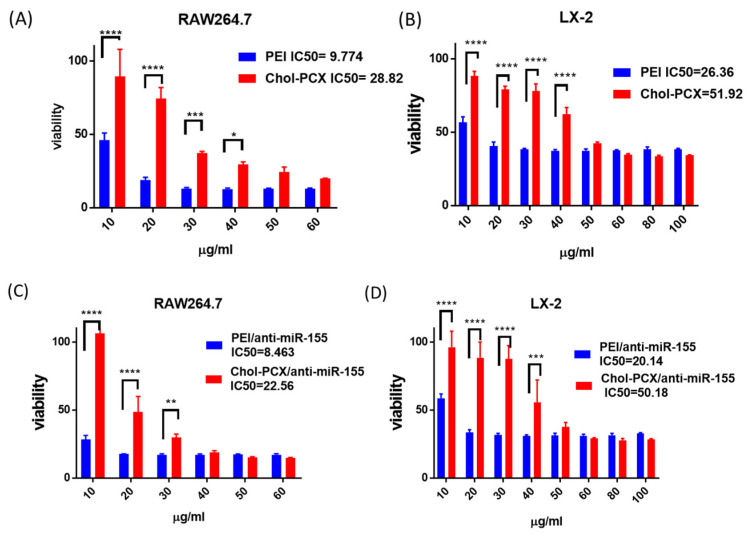
Cytotoxicity of Chol-PCX and Chol-PCX/anti-miR-155. (**A**,**B**) Cell viability of the polymers after 24 h of incubation with the macrophage RAW 264.7 and HSC LX-2. (**C**,**D**) Cell viability of Chol-PCX/anti-miR-155 after 24 h of incubation with the macrophage RAW 264.7 and HSC LX-2. Data were analyzed by one-way ANOVA followed by Tukey’s comparison test. Data are expressed with mean ± SD. * *p* < 0.05, ** *p* < 0.01, *** *p* < 0.001, and **** *p* < 0.0001.

We next investigated the cytotoxicity of Chol-PCX in human HSC LX-2 and murine macrophage RAW 264.7 (Figure 3). We used PEI as the control polymer as it has been widely used as a non-viral nucleic acid delivery vector, but its practical application has been hindered by severe cytotoxicity [40]. In both RAW 264.7 and LX-2 cells, control PEI showed strong toxicity starting from a low concentration of 20 µg/mL. However, Chol-PCX showed lower toxicity in both cell lines. These results aligned well with our previous study showing that covalent cholesterol modification decreases the cytotoxicity of polycations [39]. Moreover, we tested the cytotoxicity of Chol-PCX/anti-miR-155 and PEI/anti-miR-155 on the RAW 264.7 and LX-2 cell lines. The results showed that encapsulation of miRNA had no significant change in the cytotoxicity of the polymers (Figure 3C,D).

**Figure 4:**
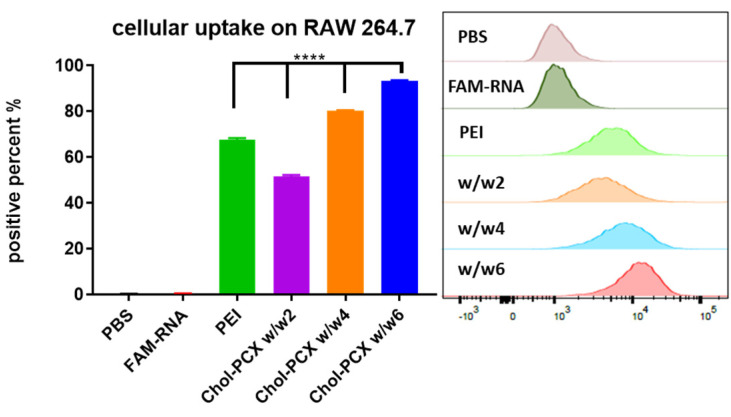
Uptake of Chol-PCX/miRNA nanoparticles after 4 h of incubation with RAW 264.7 macrophages by flow cytometry (histogram on the right). Data were analyzed by one-way ANOVA followed by Tukey’s comparison test. Data are expressed as mean ± SD (*n* = 3). **** *p* < 0.0001.

To evaluate if Chol-PCX facilitates effective intracellular uptake and trafficking for miRNA delivery, we measured cellular uptake in the macrophage cell line. We used nanoparticles prepared with fluorescently labeled FAM-RNA. Cell uptake, expressed as the % of fluorescence-positive cells, was increased from 50% to 93% as the *w*/*w* ratio of Chol-PCX/RNA increased from two to six. The uptake was comparable or higher than the control PEI (Figure 4). Nanoparticles prepared at *w*/*w* four had a relatively small size, moderately positive zeta potential, and good cellular uptake. Thus, *w*/*w* four was chosen for most subsequent in vitro and in vivo experiments.

### 3.3. Chol-PCX/anti-miR-155 Nanoparticles Downregulate miR-155 Expression in Macrophages

**Figure 5:**
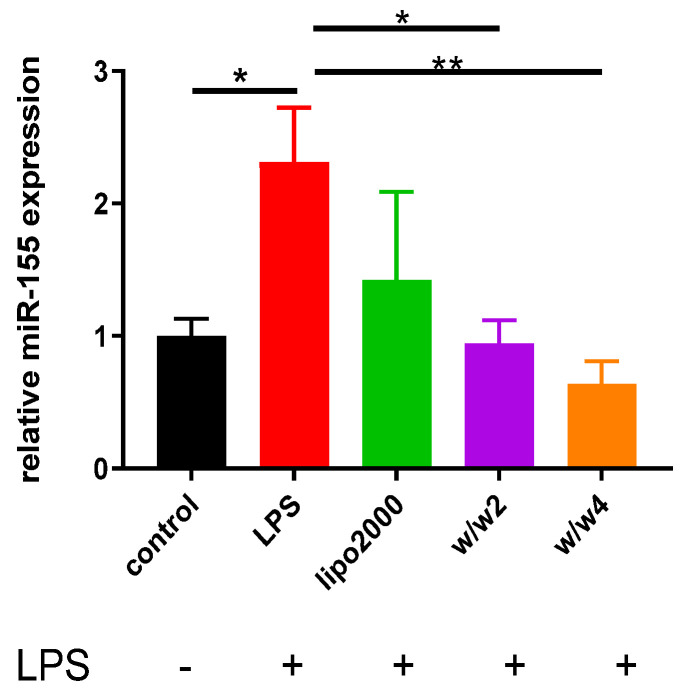
Silencing of miR-155 expression by Chol-PCX/anti-miR-155 in activated macrophages. Chol-PCX/anti-miR-155 prepared at *w*/*w* 2 and 4. Lipofectamine 2000 (lipo2000) was used as the positive control. Data were analyzed by one-way ANOVA followed by Tukey’s comparison test. Data are expressed as mean ± SD (*n* = 3). * *p* < 0.05, ** *p* < 0.01.

In vitro knockdown effects of Chol-PCX/anti-miR-155 were evaluated in the RAW 264.7 cells (Figure 5). We used LPS, an endotoxin related to the pathogenesis of AALD, to activate the macrophages and induce miR-155 overexpression [19]. The cells treated with LPS showed a two-fold increase in the expression of miR-155 over the control group. Lipofectamine 2000/anti-miR-155 was used as the positive control, and it silenced 38% of miR-155 expression. Chol-PCX/anti-miR-155 outperformed the Lipofectamine 2000 control at both *w*/*w* ratios as it decreased the miR-155 expression by 59% (*w*/*w* two) and 72% (*w*/*w* four).

### 3.4. Inhibition of CXCR4 by Chol-PCX In Vitro

**Figure 6:**
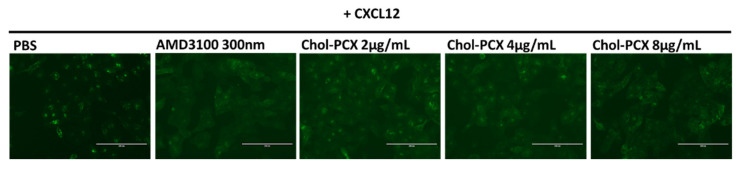
CXCR4 redistribution. U2OS-overexpressing EGFP-CXCR4 cells were treated with Chol-PCX for 30 min before incubation with 10 nM CXCL12. AMD3100 (300 nM) was used as the positive control (scale bar = 200 µm).

The CXCR4 antagonism efficacy of Chol-PCX in vitro has been tested using a CXCR4 redistribution assay (Figure 6). This assay can be applied to track and visualize the translocation of EGFP-tagged CXCR4 receptors on the cell membrane to endosomes upon CXCL12 stimulation, which is a typical behavior for G-protein-coupled receptors. As shown in Figure 6, the CXCL12-activated cells (PBS) exhibited CXCR4 translocation, shown by higher fluorescence inside the cells, while minimal fluorescence was found on the cell membrane surface. Cells that were treated with CXCR4 antagonist AMD3100 exhibited a diffused pattern of green fluorescence, indicating the inhibition of CXCR4 translocation after SDF-1 stimulation. All the Chol-PCX demonstrated strong CXCR4 inhibition. Our results confirmed that Chol-PCX functions as a CXCR4 antagonist polymer.

### 3.5. Chol-PCX Nanoparticles Biodistribution in AALD Fibrosis Model

**Figure 7:**
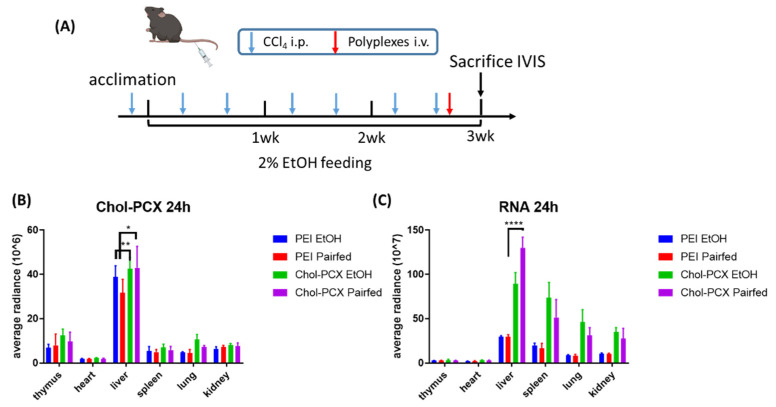
Biodistribution of Chol-PCX and PEI Cy5.5-siRNA nanoparticles. (**A**) Mice were treated with CCl_4_ for 3 weeks and fed 2% EtOH or a control diet, with the intravenous (IV) injection of the nanoparticles at the end of feeding. (**B**) Distribution in different organs 24 h after the IV injection of nanoparticles. (**C**) RNA distribution in different organs 24 h after the IV injection of nanoparticles. Data were analyzed by two-way ANOVA followed by Tukey’s comparison test. Data are expressed as mean ± SD (*n* = 6). * p < 0.05, ** *p* < 0.01, and **** *p* < 0.0001.

We investigated the biodistribution of Cy3-Chol-PCX/Cy5.5-siRNA and control Cy3-PEI/Cy5.5-siRNA by IV injection in established AALD fibrosis. The nanoparticles were injected at the end of the model and were sacrificed after 24 h (Figure 7A). The organs were harvested and imaged using IVIS. As shown in Figure 7B,C, the polymers and RNA were accumulated predominantly in the liver. Chol-PCX nanoparticles had significantly enhanced accumulation in the liver compared with PEI nanoparticles.

### 3.6. Chol-PCX/anti-miR-155 Treatment Ameliorates AALD Fibrosis

**Figure 8:**
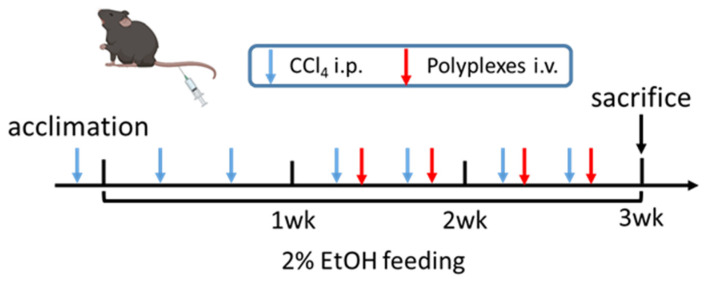
Experimental and treatment schemes. Mice were treated with CCl_4_ for 3 weeks and fed a 2% EtOH or control diet, with intravenous (IV) treatments twice per week for two weeks.

**Figure 9:**
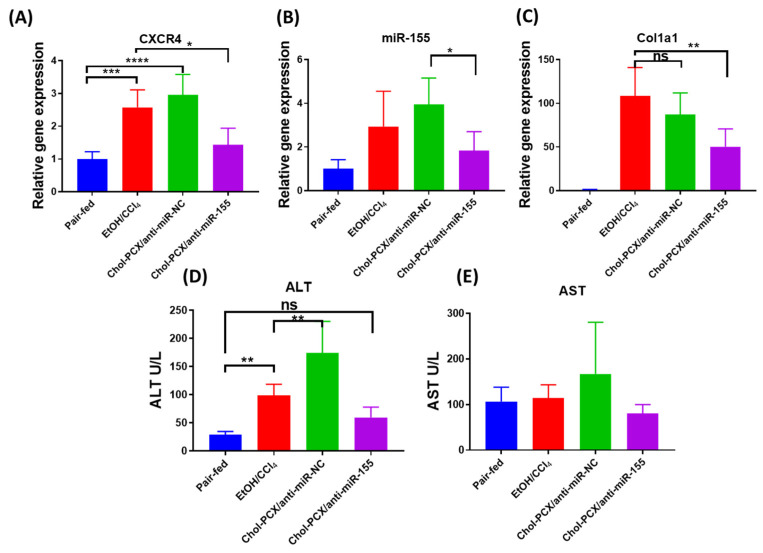
(**A**–**C**) Effect of treatment on hepatic CXCR4, Col1a1, and miR155 gene expression. (**D**,**E**) Serum ALT and AST. Data were analyzed by one-way ANOVA followed by Tukey’s comparison test. Data are expressed as mean ± SD (*n* = 6). * *p* < 0.05, ** *p* < 0.01, *** *p* < 0.001, and **** *p* < 0.0001, *ns* not significant.

To establish a model of AALD fibrosis, we put the mice on a 2% EtOH LD or isocaloric (pair-fed group) diet and combined it with a secondary liver insult achieved by injection with CCl_4_ for 3 weeks [30]. The feeding and treatment schedule is shown in Figure 8. We first validated that both of our therapeutic targets are upregulated in AALD fibrosis. The hepatic gene expression of CXCR4 increased almost 2.5-fold, and miR-155 was also elevated around 3-fold in the AALD fibrosis group (EtOH/CCl_4_) (Figure 9A,B). This was accompanied by an around 100-fold increased collagen I (Col1a1) expression in the mice treated with CCl_4_ (Figure 9C).

**Figure 10:**
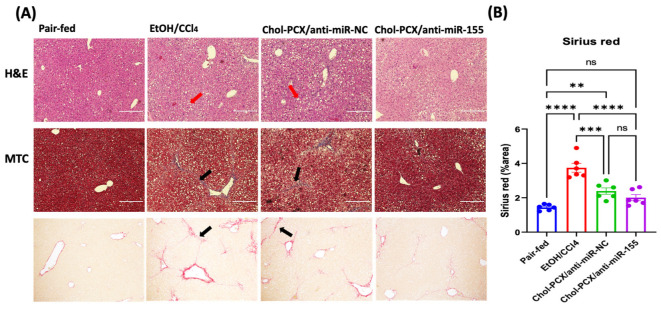
Therapeutic efficacy of Chol-PCX/anti-miR-155 in AALD fibrosis. (**A**) Hematoxylin and eosin stain (H&E), Masson’s trichrome stain (MTC), and Sirius Red stain for liver tissues in different groups. Red arrows in H&E represent fat droplets, black arrows in MTC or Sirius Red represent bridging fibrosis. (**B**) Sirius Red quantification for collagen deposition by ImageJ. Data were analyzed by one-way ANOVA followed by Tukey’s comparison test. Data are expressed by mean ± SEM. ** *p* < 0.01, *** *p* < 0.001, and **** *p* < 0.0001.

To evaluate the therapeutic efficacy, we first measured the levels of ALT and AST. ALT and AST are important biomarkers for liver injury. In AALD, EtOH usually exacerbates ALT, but AST is often not changed [30]. We found that treatment with Chol-PCX/anti-miR-155 nanoparticles significantly decreased the level of ALT and slightly decreased AST when compared with the untreated AALD fibrosis group (EtOH/CCl_4_ + PBS-injected) (Figure 9D,E). Surprisingly, ALT was elevated significantly in the Chol-PCX/anti-miR-NC group. We then examined whether anti-miR-155 delivery reduced liver fibrosis using histological analyses. The untreated AALD fibrosis liver samples showed bridging fibrosis and fat droplets that were clearly visible in the H&E and MTC slides (Figure 10A). The quantification of Sirius Red staining indicated that treatment with Chol-PCX/anti-miR-155 markedly suppressed collagen deposition compared with the untreated animals and animals treated with nanoparticles containing anti-miR-NC (Figure 10B). Collagen reduction was also verified by the expression of Col1a1 expression using RT-PCR, as shown in Figure 7C. Overall, the morphological and mRNA assessments indicate that treatment with Chol-PCX/anti-miR-155 particles results in the efficient silencing of miR-155 and CXCR4 antagonism in the AALD fibrosis livers, which is likely due to reduced KC-mediated signaling and HSC activation. Chol-PCX nanoparticles containing control anti-miR-NC showed a moderate therapeutic effect on the expression of collagen compared with Chol-PCX/anti-miR-155.

**Figure 11:**
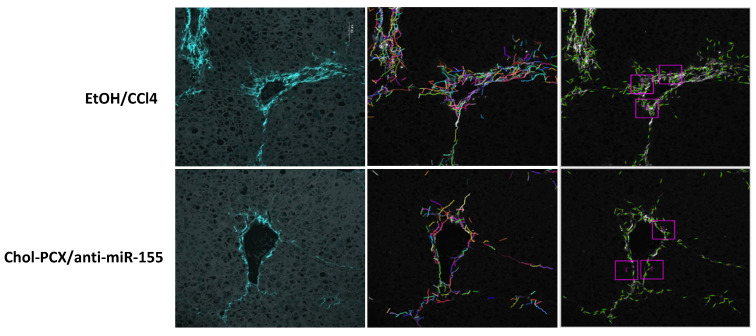
SHG imaging of liver collagen fibers. Representative images from central veins of liver samples with individual pseudocolored collagen fibers identified; pink squares show the 50 µm ROIs to calculate average collagen fiber counts, quantitative analyses of collagen organization, and content.

Further confirmation of the antifibrotic effects of the Chol-PCX/anti-miR-155 particles was performed using SHG microscopy to image collagen and its molecular organization [41]. AALD fibrosis has a pericentral vein pattern of collagen accumulation, which can be differentiated from most chronic liver diseases, where fibrosis is mainly located around portal veins [42]. Collagen fibers were imaged at 860 nm in the untreated AALD fibrosis and animals treated with the Chol-PCX/anti-miR-155 nanoparticles (Figure 11). Images were collected from central venules in a blinded fashion. We observed decreased number of fibers and decreased fiber length in the nanoparticle group relative to the untreated group. There were no additional differences or trends in collagen fiber curvature. Standardized 50 µm square ROIs were used to calculate the average collagen fiber counts at the central vein (excluding the surrounding tissue). Three independent ROIs were measured and then averaged to determine the average collagen fiber number/count and assess the alignment of those fibers relative to each other. There was no difference in the alignment, but a significantly elevated average number of collagen fibers (150 vs. 133, *p* < 0.001), fiber length (48 vs. 46.5, *p* < 0.01), and width (3.95 vs. 3.79, *p* < 0.001) associated with the 50 μm sampling ROIs at the central vein was observed for the untreated group.

### 3.7. Analysis of the Therapeutic Mechanism of Action of the Nanoparticles in AALD Fibrosis

**Figure 12:**
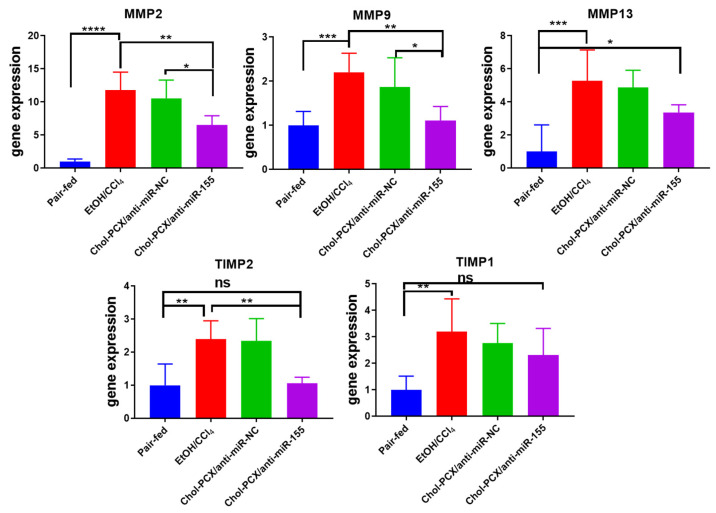
Analysis of AALD fibrosis markers. MMP and TIMP expression. Data were analyzed by one-way ANOVA followed by Tukey’s comparison test. Data are expressed as mean ± SD. * *p* < 0.05, ** *p* < 0.01, *** *p* < 0.001, and **** *p* < 0.0001, *ns* not significant.

Further analysis of the liver samples showed the enhanced expression of crucial fibrosis markers, including MMPs and TIMPs, that play an important role in extracellular matrix remodeling. It has been previously reported that an increase in these markers correlates with miR-155 expression in AALD; however, no changes were seen in miR-155 KO mice [19]. In our study, the mRNA expression levels of MMP-2, -9, and -13, as well as TIMP 1 and 2, were significantly increased in the untreated AALD fibrosis group. Treatment with Chol-PCX/anti-miR-155 particles resulted in a significant reduction in all the analyzed fibrotic markers (Figure 12).

**Figure 13:**
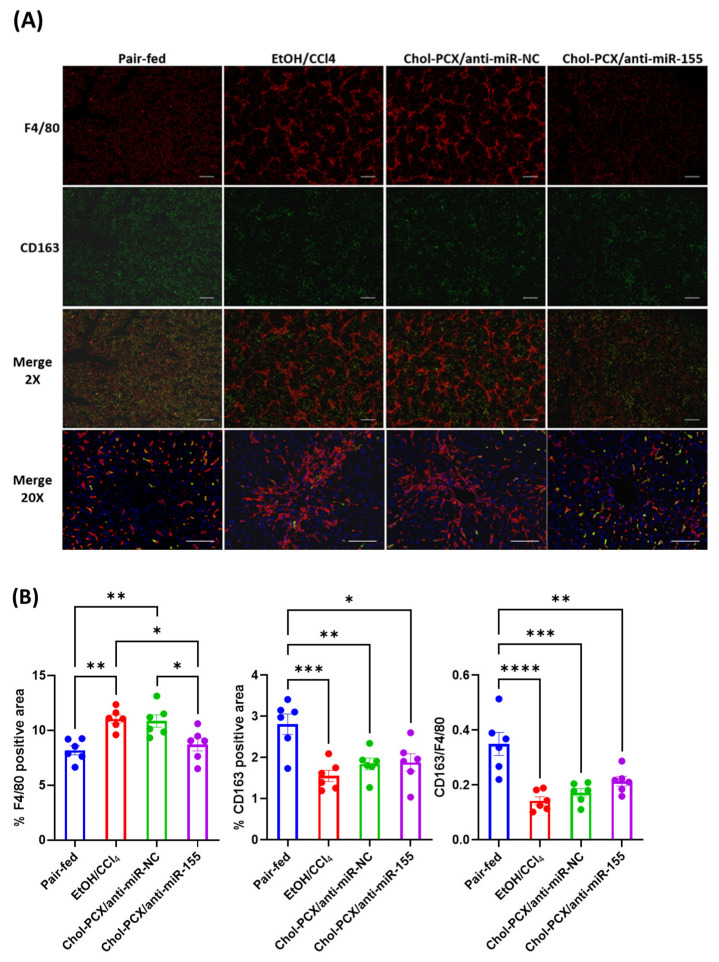
Immunohistochemistry for F4/80 (total macrophage or KCs stain) and CD163 (M2 phenotype). (**A**) Red fluorescence representing F4/80 and green fluorescence representing CD163. Scale bar: 2× (400 µm), 20× (200 µm). (**B**) Total macrophage content and CD163^+^ M2 macrophage quantification from the confocal images. Data were analyzed by one-way ANOVA followed by Tukey’s comparison test. Data are expressed as mean ± SEM (*n* = 6). * *p* < 0.05, ** *p* < 0.01, *** *p* < 0.001, and **** *p* < 0.0001.

Alcohol and associated metabolites can initiate hepatic inflammation, which contributes to the progression of AALD. An alcohol-induced increase in profibrotic macrophage infiltration was previously observed in wild-type mice compared to miR-155 KO mice [19]. In our study, we analyzed the hepatic accumulation of macrophages by F4/80 immunohistochemistry. Untreated AALD fibrosis samples (EtOH/CCl_4_ + PBS-injected) showed a “chicken wire” pattern compared with the pair-fed group (Figure 13A). Additionally, the number of F4/80^+^ macrophages in the EtOH/CCl_4_ (PBS-injected) group significantly increased when compared with the pair-fed group. The number and distribution pattern of the hepatic F4/80^+^ macrophages in mice treated with the control anti-miR-NC particles were similar to those of the untreated group. However, the Chol-PCX/anti-miR-155 group clearly had significantly reduced F4/80^+^ macrophage accumulation than that of the EtOH/CCl_4_ (Figure 13B) group, to a level comparable to the pair-fed group. Therefore, there was a significant anti-inflammatory effect in the Chol-PCX/anti-miR-155 treatment group compared with the other groups, which contributed to the reversal of AALD fibrosis. Moreover, the CD163^+^ macrophages decreased in the AALD fibrosis group, indicating decreased M2 polarization in this model. In the Chol-PCX/anti-miR-155 group, there was a slight increase in the CD163^+^-to-F4/80^+^ ratio compared with the untreated group, suggesting the start of recovery of M2-polarized macrophages after treatment (Figure 13B). The observed increase in M2 macrophages does not account for the changes observed in the total macrophages. The nanoparticles are thus likely decreasing the M1 macrophages, which contributes to the decrease in total macrophages in the Chol-PCX/anti-miR-155 treatment group.

**Figure 14:**
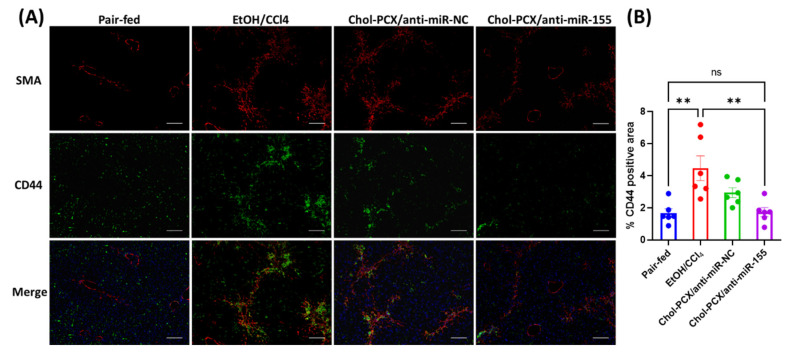
Immunohistochemistry for α-SMA (activated HSCs stain) and CD44. (**A**) Red fluorescence representing α-SMA and green fluorescence representing CD44. Scale bar = 100 µm. (**B**) CD44-positive area quantification from the confocal images. Data were analyzed by one-way ANOVA followed by Turkey’s comparison test. All data are shown as mean ± SEM (*n* = 6). ** *p* < 0.01, *ns* not significant.

The activation of HSCs in AALD is marked by the overexpression of CD44 receptors. CD44 is highly expressed in tumors but not very common in healthy livers. During fibrosis, however, the proliferation of HSCs is accompanied by increased CD44 expression [43,44]. Here, we illustrate the effect of the nanoparticle treatment on CD44 expression in the liver. α-SMA immunohistochemistry was used for staining the activated HSCs. It was shown that the untreated EtOH/CCl_4_ group had a significant increase in the number of activated HSCs compared to the pair-fed group. The activation of HSCs was markedly reduced in the Chol-PCX/anti-miR-155 treatment group (Figure 14A). CD44 expression also significantly increased in the untreated EtOH/CCl_4_ group, and it was at least partly colocalized with α-SMA, confirming expression in activated HSCs. However, a significant amount of CD44 expression did not overlap with α-SMA, also indicating its presence in macrophages and other immune cells. In the Chol-PCX/anti-miR-155 group, CD44 expression was significantly lowered when compared with the untreated EtOH/CCl_4_ group. The percent of the CD44-positive area was quantified, and the results are shown in Figure 14B. Treatment with Chol-PCX/anti-miR-155 completely reversed the increase in CD44 expression across all the cell types observed in untreated AALD fibrosis.

## 4. Discussion

To test our hypothesis that CXCR4-inhibiting polymers can deliver therapeutic miRNA in a model of moderate alcohol consumption with secondary liver insult as a new approach to combination treatment, we first synthesized Chol-PCX and evaluated its transfection efficacy in vitro and therapeutic effects in vivo. We focused our studies on the overall efficacy of the combination treatment and on dissecting the contributions of CXCR4 inhibition and miR-155 silencing on the effectiveness of the treatment.

We first examined the role of CXCR4 inhibition. Non-parenchymal liver cells are crucial players in AALD fibrogenesis. HSCs differentiate and proliferate into activated HSCs, while KCs secrete proinflammatory cytokines. These two cell types communicate with other hepatic cells to accelerate the progression of fibrosis [6]. The inhibition of the CXCR4 axis in activated HSCs has been previously used successfully in the treatment of liver fibrosis [45,46]. However, there are also studies suggesting that the effect of CXCR4 inhibition depends on the degree of liver injury. For example, in an acute liver injury and early chronic liver fibrosis model, mice treated with a CXCR4 antagonist, AMD3100, expressed higher hepatic α-SMA and showed increased hepatic collagen content and fibrosis over untreated controls. This suggested that CXCR4 inhibition worsens hepatic injury in early stage disease [47]. The CXCR4 ligand CXCL12 has previously been shown to have a potentially protective role during hepatic fibrosis through the expansion of hepatic progenitor and oval cells [48], which are beneficial for liver regeneration. HSCs were activated and the number of circulating neutrophils increased in the liver of mice receiving AMD3100 at the onset of liver injury [49]. Our study confirms the complex role of CXCR4 in the early stages of AALD fibrosis. On the one hand, CXCR4 inhibition alone (animals treated with Chol-PCX/anti-miR-NC) decreased hepatic collagen content (Figure 10B, *p* = 0.0003), but there was no effect on the α-SMA expression observed in other studies with similar polymers. On the other hand, the liver injury marker ALT was elevated in animals treated with CXCR4 inhibition alone. Our results are consistent with previous studies on liver fibrosis and expand them to AALD to suggest similarly complex effects of CXCR4 inhibition on the AALD fibrosis liver microenvironment.

In contrast to CXCR4, miR-155 proved to be a valid therapeutic target in AALD fibrosis. miR-155 was strongly upregulated in AALD livers, and its silencing with the Chol-PCX/anti-miR-155 nanoparticles resulted in overall therapeutic benefit. We have observed multiple effects of miR-155 silencing, including a decrease in the hepatic expression of CXCR4 and CD44. To explain this finding, we have to look into the initial stages of the liver injury, when KCs trigger the recruitment of additional immune cells. During AALD pathogenesis, LPS binds to CD14/TLR4 on KCs, causing nuclear factor-kappa beta (NF-ĸB) activation, which in turn leads to the KC secretion of a series of cytokines and chemokines, such as transforming growth factor-β (TGF-β) and tumor necrosis factor-α (TNF-α) [50]. The inflammatory factor TGF-β is one of the most widely recognized and powerful profibrogenic mediators, and promotes the accumulation of ECMs by HSCs [23]. The cytokines released by activated KCs damage hepatocytes and induce HSC activation [51]. The downregulation of miR-155 contributes to the activation of HSC through regulating the EMT process and the ERK1 pathway [52]. CXCL12/CXCR4 is also known to activate the ERK1/2 cascade to induce the proliferation of HSCs in liver fibrosis [23]. We believe that there is a communication between HSCs and KCs that is partly regulated by miR-155 and CXCR4, and that participates in the regulation of the overlapping pathways during the progression and reversal of AALD fibrosis. Treatment with the Chol-PCX/anti-miR-155 nanoparticles reduces a series of inflammatory factors from the activated KCs, causing the reduced activation of HSCs and CXCR4 as well as CD44 expression. This is further supported by our finding that the inflammatory F4/80^+^ macrophages were significantly reduced in the treated group, while the CD163^+^ M2 macrophages were increased as a result of the treatment.

## 5. Conclusions

In conclusion, silencing miR-155 expression may serve as a potential treatment of AALD-associated liver fibrosis. The therapeutic role of CXCR4 inhibition in liver fibrosis deserves further investigation.
